# Direct anterior approach (DAA) vs. conventional approaches in total hip arthroplasty: A RCT meta-analysis with an overview of related meta-analyses

**DOI:** 10.1371/journal.pone.0255888

**Published:** 2021-08-24

**Authors:** Philip Lazaru, Simon Bueschges, Nikolai Ramadanov

**Affiliations:** 1 Center for Surgery, Evangelical Hospital Ludwigsfelde-Teltow, Ludwigsfelde, Germany; 2 Faculty of Medicine, Department of Statistics, University of Salamanca, Salamanca, Spain; 3 Department of Emergency Medicine, University Hospital Jena, Friedrich Schiller University, Jena, Germany; Azienda Ospedaliero Universitaria Pisana, ITALY

## Abstract

**Objectives:**

Several systematic reviews and meta-analyses on short-term outcomes between total hip arthroplasty (THA) through direct anterior approach (DAA) compared to THA through conventional (including anterior, anterolateral, lateral transgluteal, lateral transtrochanteric, posterior, and posterolateral) approaches (CAs) in treatment of hip diseases and fractures showed contradicting conclusions. Our aim was to draw definitive conclusions by conducting both a fixed and random model meta-analysis of quality randomized controlled trials (RCTs) and by comparison with related meta-analyses.

**Design:**

We performed a systematic literature search up to May 2020 to identify RCTs, comparing THA through DAA with THA through CAs and related meta-analyses. We conducted risk of bias and level of evidence assessment in accordance with the Cochrane’s Risk of Bias 2 tool and with the guidelines of the Centre for Evidence-Based Medicine. We estimated mean differences (MD) with 95% confidence intervals (CI) through fixed and random effects models, using the DerSimonian and Laird method. Heterogeneity was assessed using tau-square (τ^2^). Our conclusions take into account the overall results from related meta-analyses.

**Results:**

Nine studies on THA through DAA met the criteria for final meta-analysis, involving 998 patients. Three studies were blinded RCTs with a level I evidence, the other 6 studies were non-blinded RCTs with a level II evidence. We came to the following results for THA through DAA compared to THA through CAs: operation time (I^2^ = 92%, p<0.01; fixed: MD = 15.1, 95% CI 13.1 to 17.1; random: MD = 18.1, 95% CI 8.6 to 27.5); incision length (I^2^ = 100%, p<0.01; fixed: MD = -2.9, 95% CI -3.0 to -2.8; MD = -1.1, 95% CI -4.3 to 2.0); intraoperative blood loss (I^2^ = 87%, p<0.01; fixed: MD = 51.5, 95% CI 34.1 to 68.8; random: MD = 51.9, 95% CI -89.8 to 193.5); VAS 1 day postoperatively (I^2^ = 79%, p = 0.03; fixed: MD = -0.8, 95% CI -1.2 to -0.4; random: MD = -0.9, 95% CI -2.0 to 0.15); HHS 3 months postoperatively (I^2^ = 52%, p = 0.08; fixed: MD = 2.8, 95% CI 1.1 to 4.6; random: MD = 3.0, 95% CI -0.5 to 6.5); HHS 6 months postoperatively (I^2^ = 0%, p = 0.67; fixed: MD = 0.9, 95% CI -1.1 to 2.9; random: MD = 0.9, 95% CI -1.1 to 2.9); HHS 12 months postoperatively (I^2^ = 0%, p = 0.79; fixed: MD = 0.7, 95% CI -0.9 to 2.4; random: MD = 0.7, 95% CI -0.9 to 2.4). We compared our findings with 7 related meta-analyses.

**Conclusions:**

Considering the results of our meta-analysis and the review of related meta-analyses, we can conclude that short-term outcomes of THA through DAA were overall better than THA through CAs. THA through DAA had a shorter incision length, a tendency towards a lower pain VAS 1 day postoperatively and better early postoperative functional outcome than THA through CAs. The intraoperative blood loss showed indifferent results. THA through DAA had a longer operation time than THA through CAs.

## Introduction

Total hip arthroplasty (THA) can effectively relieve pain and restore function in patients with advanced hip osteoarthritis [1]. Hip replacement surgery is also preferred for displaced femoral neck fractures in elderly patients [2,3], as head-preserving procedures show a high complication rate [4–6]. There are six conventional surgical approaches to the hip joint: anterior, anterolateral, lateral transgluteal, lateral transtrochanteric, posterior, and posterolateral approach. Minimally invasive approaches are modifications of conventional approaches (CAs), introduced with the aim of achieving a better patient outcome through muscle-sparing techniques and shorter incision lengths [7–19]. Among approaches to the hip joint, the direct anterior approach (DAA) showed potential to highlight as beneficial [10,12,13,20]. DAA follows internervous and intermuscular planes, namely the anatomical interval between the muscles of Sartorius and tensor fasciae latae, and therefore leads to less soft tissue trauma [21–23]. DAA to the hip joint was introduced in 1881 by the German surgeon Carl Hueter [12]. Smith-Petersen popularized DAA in 1917 with a description in English-language literature [24]. Judet reported in 1985 on the method using a traction (fracture) table (TT) [25].

Over the past decade, several systematic reviews and meta-analyses were conducted to reveal differences in outcomes between THA through DAA compared to THA through CAs [26–34]. Unfortunately, their conclusions were partly contradicting. While some of those meta-analyses found an advantage of THA through DAA compared to other approaches [27–29,31–33], the other meta-analyses came to different conclusions [26,30,34]. Furthermore, only one of those systematic reviews and meta-analyses considered the TT utilization in THA through DAA [28]. Science needs further high quality research on this subject in order to be able to draw a definitive conclusion.

Our first objective was to compare short-term outcomes of THA through DAA and THA through CAs in treatment of hip diseases and fractures by performing systematic literature review and both a fixed and random model meta-analysis of quality RCTs. Our second objective was to compare our results with related meta-analyses in order to draw definitive conclusions.

## Materials and methods

### Reporting guidelines and protocol registration

We followed the Preferred Reporting Items for Systematic Reviews and Meta-Analysis-Protocols (PRISMA-P) guidelines [35]. The review protocol was registered retroactively with the International Prospective Register of Systematic Reviews (PROSPERO) on 28 January 2021 and finally approved on 28 February 2021 (CRD42021233481) at http://www.crd.york.ac.uk/PROSPERO/

### Search methodology

We built a BOOLEAN search strategy (see S1 Appendix) and adapted it to the syntax of the used databases. Results of the searches were exported to a reference management software [36]. Our search was performed in the following databases: PubMed, Google Scholar, The Cochrane Library, Clinical trials. Furthermore, we checked citations of screened studies and reviews for additional records. The search continued up to May 2020. We searched those databases for related meta-analyses and checked their references for relevant studies.

### Study selection

The process was performed in two stages. Two independent reviewers (NR and PL) screened titles and abstracts to identify articles for further consideration. The full text of the selected articles were obtained and screened again by the two reviewers (NR and PL) according to inclusion criteria. Disagreements were resolved by consensus. Kappa coefficient was used to measure the agreement between the reviewers.

### Inclusion criteria and outcomes

Inclusion criteria were: randomized controlled trials (RCTs) with no restriction to language and publication date; studies which compared outcomes in THA through DAA and THA through CAs. We included human participants with hip disease or hip fracture. We did not include studies comparing outcomes in THA through DAA and THA through mini-incision approaches as well as surgical techniques using of a computer navigation system. In the studies concerned, we relied on the authors’ assessment that a mini-incision approach was used. The types of measured outcomes were:

1. **Surgical outcomes:**
  - The **operation time** in min. was defined as the period of time from the beginning of skin incision to surgical closure.
  - The **incision length** in cm was measured on graduated scale.
  - The **intraoperative blood loss** in ml was the total amount of blood from the suction device.
2. **Pain Visual Analogue Scale**
  - The **pain Visual Analogue Scale (VAS)** was an instrument for measuring pain intensity, providing a range of scores from 0 to 10 points [37,38].
3. **Functional outcome:**
  - The **Harris Hip Score (HHS)** was developed for assessment of the results of hip surgery [39]. The hip joint function was evaluated at periodically time intervals after operation. The score collects points from the assessment of four aspects: pain, function, degree of deformity and range of motion of the hip. The higher the added score, the better the results, providing a range of added scores from 0 to 100 points.
4. **Radiological outcomes:**
  - The prosthesis **cup abduction** angle and the **anteversion** angle have ideal values for positioning: abduction angle from 40° to 50° and anteversion angle from 10° to 25° [40]. Especially, the ideal cup anteversion is of great importance, since a too large angle often leads to anterior dislocation and a too small angle often leads to posterior dislocation.

### Data extraction and analysis

We extracted data on study characteristics, methods, quality assessment, on characteristics of participants, on details of the interventions, and on measured outcomes into a standard electronic spreadsheet and the Cochrane software program Review Manager Version 5.3 [41]. We contacted the authors for missing data. In some cases relevant data was still missing, so the corresponding study was excluded in order to guarantee a high quality inclusion of RCTs.

### Risk of bias and level of evidence

Our risk of bias and level of evidence assessment were performed in accordance with the Cochrane’s Risk of Bias 2 (RoB 2) tool [42] and with the guidelines of the Centre for Evidence-Based Medicine (Oxford, UK) [43]. Furthermore, we considered the publication year in risk of bias assessment, since it was shown that publication bias is smaller in meta-analyses of more recent studies [44].

### Statistical analysis

#### Measures of treatment effect

DAA represented the “experimental group” and CAs represented the “control group”. Mean differences (MDs) with 95% confidence intervals (CIs) were estimated through fixed and random effects models for all outcomes. A common τ^2^ was assumed for calculation of the random effects estimates, using the DerSimonian and Laird method. Study weighting was performed by inverse variance [45]. We evaluated the results and analysed them on basis of the Cochrane Handbook for Systematic Reviews of Interventions [46], R packages meta [47] and metafor [48].

#### Assessment of heterogeneity

We did not pool study data that were clinically too diverse. Heterogeneity was assessed using tau-square (τ^2^), which followed a distribution with k-degrees of freedom (p value < 0.10 is indicative of heterogeneity), and a Higgins’ test I^2^ (low heterogeneity, < 25%; moderate heterogeneity, 25–75%; and high heterogeneity, > 75%) [49].

#### Interpretation of our results and comparison between our study and related meta-analyses

Our meta-analysis offers both a fixed and a random effects model which obviously increases the statistical value and enriches our study. Nevertheless, in interpretation of our results we stuck to the common scientific understanding that random effects models are more conservative and provide better estimates with wider confidence intervals [50,51]. Wherever both effects models offered different results, the random effects model was preferred. In addition, a simple comparison of our results with the results of the related meta-analyses was performed. In case that overall results of all meta-analyses cumulated in the same direction, we tried to draw a definitive conclusion. *So*, *our conclusions take into account the overall results from all meta-analyses*.

#### TT-subgroup analysis

We performed a TT subgroup-analysis in order to examine whether the utilization of a TT in THA through DAA leads to different short-term outcomes.

## Results and discussion

### Study identification and selection

**Fig 1:**
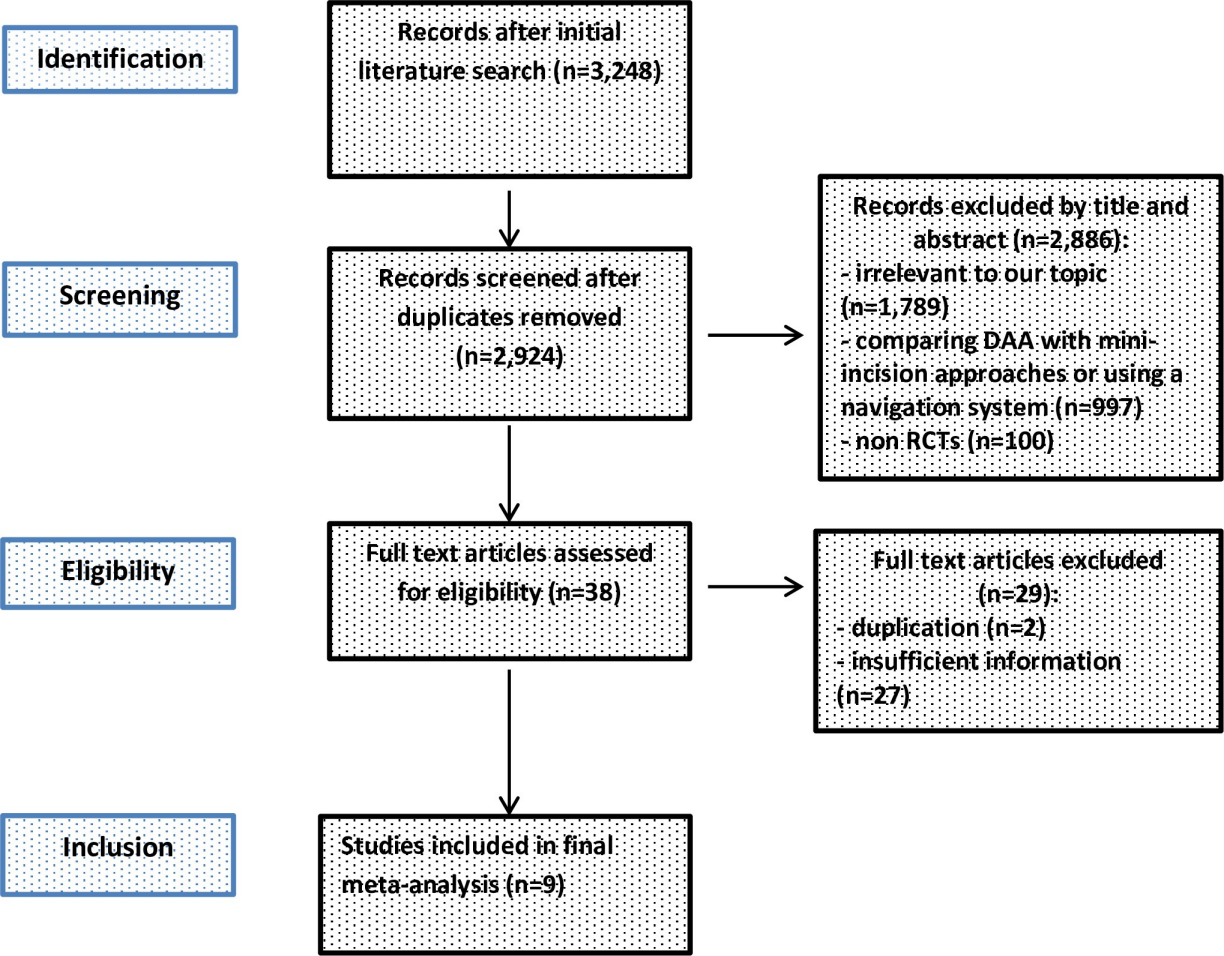
PRISMA flow diagram of the search results and selection according to our inclusion criteria. DAA, direct anterior approach; RCTs, randomized controlled trials.

We searched PubMed, Google Scholar, The Cochrane Library, Clinical trials and found 3,248 studies. Additional records were not found during manual searches of reference lists. We removed 324 duplicates, resulting in a total of 2,924 studies in initial literature search. 38 studies were assessed for eligibility after first screening procedure by title and abstract (κ = 0.95) with disagreement between the reviewers concerning 2 studies. The remaining 38 studies were read in full, and 29 were excluded according to inclusion criteria (κ = 1.0). Four of those studies were excluded because they did not provide any information on standard deviation of the outcome parameters examined [52–55]. A total of 9 studies on THA through DAA met the criteria for final meta-analysis [56–64]. Details of study identification, screening, and selection are given in a PRISMA flow diagram (Fig 1).

### Characteristics of the RCTs

Table 1 gives an overview of the main characteristics of the 9 included RCTs. The main preoperative diagnoses were osteoarthritis, femoral neck fracture and avascular necrosis of the femoral head. The 9 studies, comparing T’HA through DAA with THA through CAs, were published between 2009 and 2019, altogether involving 998 patients (with 1002 operated hip joints). 440 were operated through DAA and 558 through CAs. The sample size of the 9 studies ranged from 46 to 169 patients and they were published in English language. Of the 9 studies, 4 included conventional THA through posterolateral approach [56,57,63,64], 5 through lateral transgluteal approach [58–62]. Two studies reported to have used TT in THA through DAA [56,57]. Only one study included patients with bilateral THA [64].

**Table 1:** Main characteristics of RCTs included in network meta-analysis.

	Sample Size, n		Surgical approach		Mean Age, y (SD or range)		Gender (M/F), n		BMI, kg/m^2^ (SD or range)	
Study	Pts	Hips	DAA	CAs	DAA	CAs	DAA	CAs	DAA	CAs
**Barrett 2013 [56]**	87	87	43TT	44 pl	61,4±9,2	53,2±7,7	29/14	19/25	30,7±5,4	29,1±5
**Bon 2019 [57]**	100	100	50TT	50pl	67,26±10	68,98±7,93	21/29	23/27	26,46±3,58	26,69±3,12
**D’Arrigo 2009 [58]**	169	169	20	149 l	64±8	65±9,8	12/8	81/68	22,7±1,5	28±1,8
**De Anta-Diaz 2016 [59]**	99	99	49	50 l	63,5±12,5	64,8±10,1	26/23	26/24	26,9±3,1	26,6±3,9
**Mjaaland 2015 [60]**	163	163	83	80 l	67,2±8,6	65,6±8,6	25/58	30/50	3,6±1,9	27,6±3,9
**Nistor 2017 [61]**	70	70	35	35 l	67	64	26/9	16/19	27,45±3,76	38,63±3,12
**Reichert 2018 [62]**	148	148	77	71 l	63,2±8,2	61,9±7,8	45/32	71/0	28,1±3,7	28,3±3,4
**Rykov 2017 [63]**	46	46	23	23 pl	62,8±6,1	60,2±8,1	8/15	11/12	29±5,6	29,3±4,8
**Zhao 2017 [64]**	116	120	60	56 pl	64,9±12,1	62,2±14,7	24/36	22/34	24,35±3,1	25,58±2,83

DAA, direct anterior approach; TT, traction table; CAs, conventional approaches; pl, posterolateral approach; p, posterior approach; l, lateral approach; Pts, patients.

### Risk of bias and level of evidence

Three of 9 studies were rated with a low risk of bias [57,60,64], 3 studies with a moderate risk of bias [59,61,62] and 3 studies with a high risk of bias [56,58,63]. Table 2 shows the summarized risk of bias assessment. Three out of 9 studies were blinded RCTs with a level I evidence [57,60,64], the other 6 studies were non-blinded RCTs with a level II evidence [56,58,59,61–63].

**Table 2:** Risk of bias assessment.

Study ^[ref]^	Publication year	Random sequence generation	Allocation concealment	Blinding	Complete outcome data	No selective reporting	No other sources of bias	Overall risk of bias
**Barrett 2013 [56]**	N	Y	N	N	U	Y	U	High RB
**Bon 2019 [57]**	Y	Y	Y	Y	Y	Y	Y	Low RB
**D’Arrigo 2009 [58]**	N	Y	Y	U	Y	Y	U	High RB
**De Anta-Diaz 2016 [59]**	Y	Y	Y	U	Y	Y	Y	Moderate RB
**Mjaaland 2015 [60]**	Y	Y	Y	Y	Y	Y	Y	Low RB
**Nistor 2017 [61]**	Y	Y	Y	U	Y	Y	U	Moderate RB
**Reichert 2018 [62]**	Y	Y	Y	U	Y	Y	U	Moderate RB
**Rykov 2017 [63]**	Y	Y	Y	N	Y	Y	U	High RB
**Zhao 2017 [64]**	Y	Y	Y	Y	Y	Y	Y	Low RB

DAA, direct anterior approach; Y, Yes, positive; U, Unclear; N, No, negative; RB, risk of bias.

### Related systematic reviews and meta-analyses

The characteristics of the corresponding systematic reviews and meta-analyses are listed in Table 3. They were published from 2015 to 2019, they included 9 to 88 studies. One of them was a systematic review and network meta-analysis [30], 6 systematic reviews and meta-analyses [26–29,31,32], and 2 systematic reviews [33,34]. The results of short-term outcomes of THA through DAA compared to other approaches by the related meta-analyses are listed in Table 4.

**Table 3:** Main characteristics of the related systematic reviews and meta-analyses.

First author ^[ref]^	Publication year	Study design	Patients or hips (N)	Studies included	Major limitations
Higgins et al. [26]	2015	Meta-analysis	2,302	17 studies: 2 RCTs, 10 retrospective studies, and 5 non-randomized prospective studies	inclusion of studies with a high risk of bias and a low level of evidence
Jia et al. [27]	2019	Meta-analysis	7,377	20 studies: 4 RCTs, 13 retrospective studies, and 3 non-randomized retrospective studies	Inclusion of studies with a high risk of bias and a low level of evidence
Kucukdurmaz et al. [28]	2019	Meta-analysis	1,661	18 RCTs	navigated THA and THA through mini-incision approaches were pooled in one group with THA through CAs; two relevant recent RCTs were not included
Miller et al. [29]	2018	Meta-analysis	1,044	13 prospective studies	only 7 RCTs and a short follow-up period of 90 days postoperatively; some of the outcome parameters were pooled from studies that measured those parameters at different times
Putananon et al. [30]	2018	Network Meta-analysis	1,201	14 RCTs	limited to three outcomes: VAS, HHS, complications; posterior and posterior-2 incision approaches were pooled based on only two studies
Wang et al. [31]	2018	Meta-analysis	752	9 RCTs	included only THA from posterior approach out of CAs
Yue et al. [32]	2015	Meta-analysis	4,901	12 studies: 2 RCTs and 10 were non-randomized studies	inclusion of studies with a high risk of bias and a low level of evidence
Kyriakopoulos et al. [33]	2018	Systematic review		88 studies	no meta-analysis
Meermans et al. [34]	2017	Systematic review		42 studies	no meta-analysis

CAs, conventional approaches; HHS, Harris Hips Score; RCTs, randomized controlled trial; THA, total hip arthroplasty; VAS, Visual Analogue Scale.

**Table 4:** Short-term outcomes of THA through DAA compared to other approaches by related meta-analyses.

**Operation time**	**Results**
Our study	
fixed effects model	15.5 min. longer than CAs
random effects model	19.1 min. longer than CAs
Higgins et al. [26]	No difference
Jia et al. [27]	13 min. longer than posterior approach
Kucukdurmaz et al. [28]	7 min. longer than other approaches
Wang et al. [31]	No difference
Yue et al. [32]	8 min. longer than lateral approach
**Incision length**	**Results**
Our study	
fixed effects model	2,9 cm shorter than CAs
random effects model	No difference
Kucukdurmaz et al. [28]	3.2 cm shorter than other approaches
Wang et al. [31]	3.5 cm shorter than posterior approach
**Intraoperative blood loss**	**Results**
Our study	
fixed effects model	51.5 ml higher than CAs
random effects model	No difference
Higgins et al. [26]	No difference
Wang et al. [31]	67 ml lower *postoperative* blood loss than posterior approach
Yue et al. [32]	No difference in postoperative blood *transfusion rates* compared to lateral approach
**Pain VAS 1 day postoperatively**	**Results**
Our study	
fixed effects model	0.8 point lower than CAs
random effects model	No difference
Kucukdurmaz et al. [28]	1.3 points lower than other approaches
Miller et al. [29]	0.4 point lower than posterior approach
Putananon et al. [30]	0.1 point higher than lateral approach
Wang et al. [31]	0.7 point lower than posterior approach
**HHS postoperatively**	**Results**
**HHS 3 months postoperatively**	
Our study	
fixed effects model	2.8 points higher than CAs
random effects model	No difference
**HHS 6 months postoperatively**	
Our study	
fixed effects model	No difference
random effects model	No difference
**HHS 12 months postoperatively**	
Our study	
fixed effects model	No difference
random effects model	No difference
Higgins et al. [26]	Several functional outcomes 1.5–3 months postoperatively with tendency towards better results DAA vs. posterior approaches
Kucukdurmaz et al. [28]	5.6 points higher HHS 1.5 months postoperatively for DAA vs. other approaches; better results in WOMAC score for DAA vs. other approaches
Miller et al. [29]	1.5 months postoperatively: 0.3 point higher HHS for DAA vs. posterior approach
Putananon et al. [30]	1–1.5 months postoperatively: 2.6 points higher HHS for DAA vs. lateral approach, 4.8 points higher HHS for DAA vs. posterior approach, 10.8 higher HHS for DAA vs. posterior-2 incision approach
Wang et al. [31]	0.5 and 1.5 months postoperatively: 7.4 and 6.8 points higher HHS for DAA vs. posterior approach
Yue et al. [32]	Several functional outcomes 1.5 months up to years postoperatively: overall better results for DAA vs. lateral approach

CAs, conventional approaches; HHS, Harris Hips Score; VAS, Visual Analogue Scale; WOMAC, Western Ontario and McMaster Universities Arthritis Index.

### Outcomes

#### 1. Surgical outcomes

**Fig 2:**
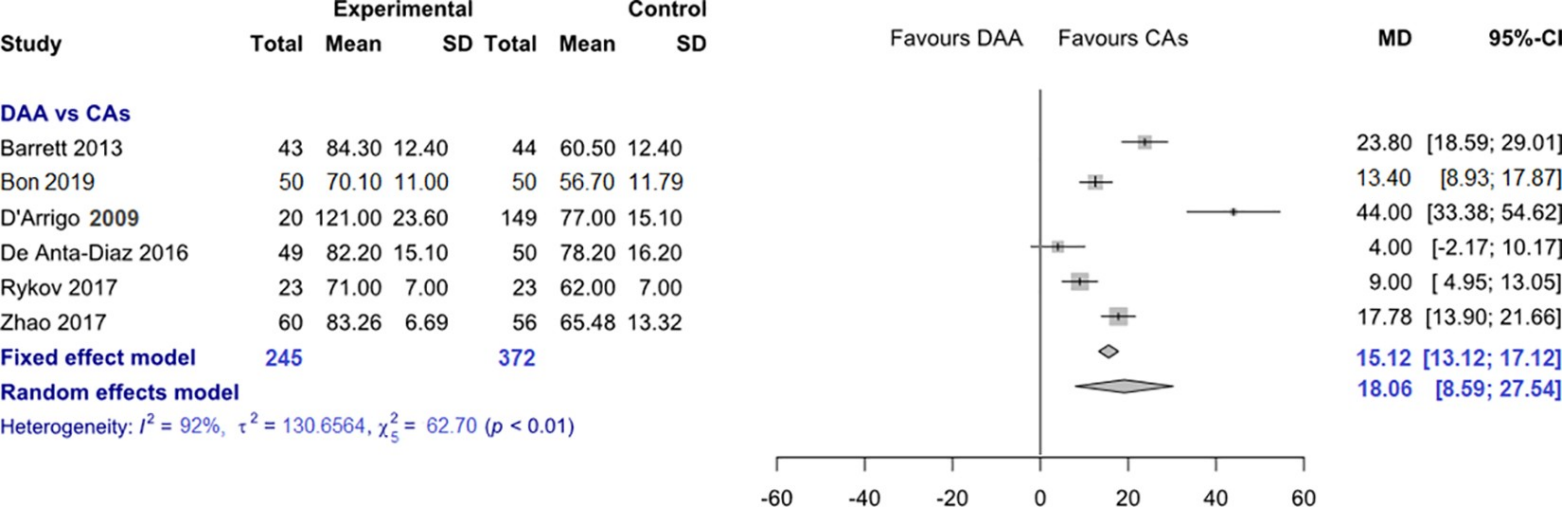
Comparison of the operation time in min. DAA, direct anterior approach; CAs, conventional approaches; SD, standard deviation; MD, mean difference; CI, confidence interval.

*Operation time*. Data on 617 patients were pooled from 6 RCTs (I^2^ = 92%, p<0.01, Fig 2). The operation time of THA through DAA was 15.1 min. longer than the operation time of THA through CAs, using a fixed effects model (MD = 15.1, 95% CI 13.1 to 17.1). The operation time of THA through DAA was 18.1 min. longer than the operation time of THA through CAs, using a random effects model (MD = 18.1, 95% CI 8.6 to 27.5).

**Fig 3:**
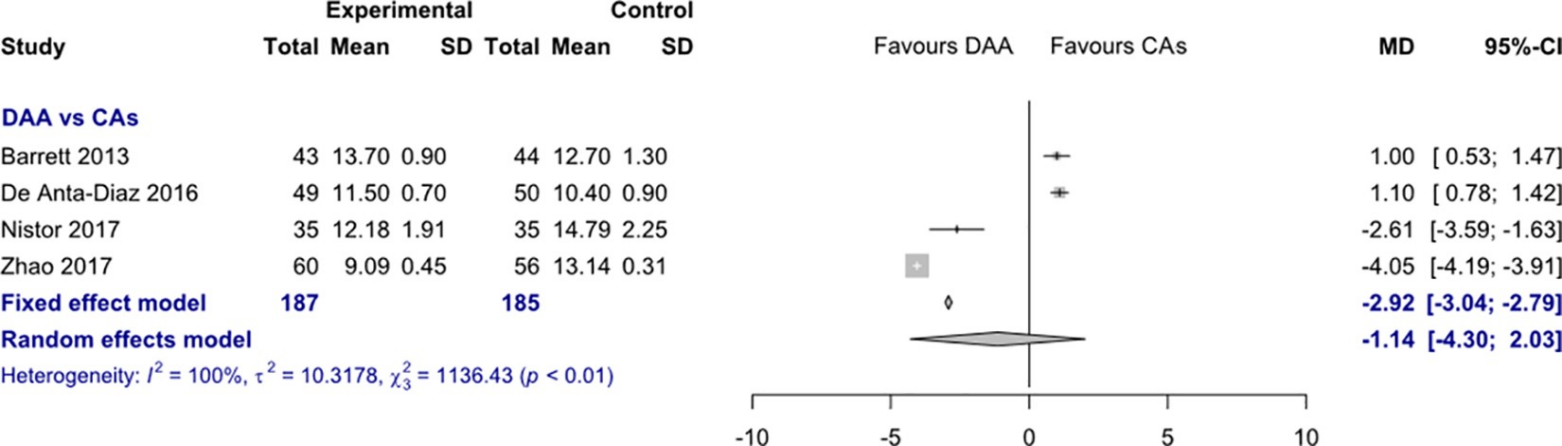
Comparison of the incision length in cm. DAA, direct anterior approach; CAs, conventional approaches; SD, standard deviation; MD, mean difference; CI, confidence interval.

*Incision length*. Data on 372 patients were pooled from 4 RCTs (I^2^ = 100%, p<0.01, Fig 3). The incision length of THA through DAA was 2.9 cm shorter than the incision length of THA through CAs, using a fixed effects model (MD = -2.9, 95% CI -3.0 to -2.8). There was no difference in incision length, using a random effects model (MD = -1.1, 95% CI -4.3 to 2.0).

**Fig 4:**
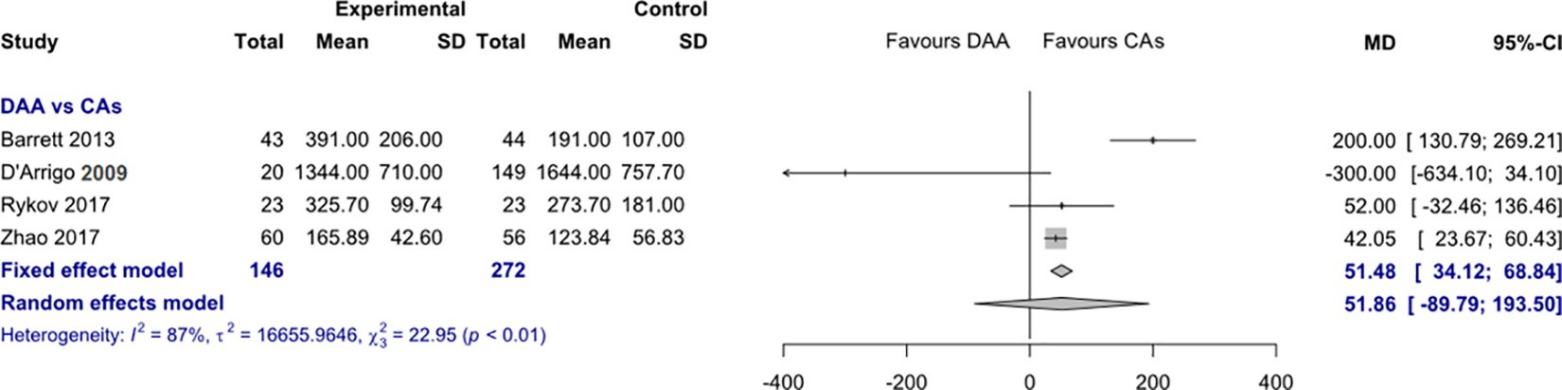
Comparison of the intraoperative blood loss in ml. DAA, direct anterior approach; CAs, conventional approaches; SD, standard deviation; MD, mean difference; CI, confidence interval.

*Intraoperative blood loss*. Data on 418 patients were pooled from 4 RCTs (I^2^ = 87%, p<0.01, Fig 4). The intraoperative blood loss of THA through DAA was 51.5 ml higher than the intraoperative blood loss of THA through CAs, using a fixed effects model (MD = 51.5, 95% CI 34.1 to 68.8). There was no difference in intraoperative blood loss, using a random effects model (MD = 51.9, 95% CI -89.8 to 193.5).

#### 2. Pain visual analogue scale

**Fig 5:**
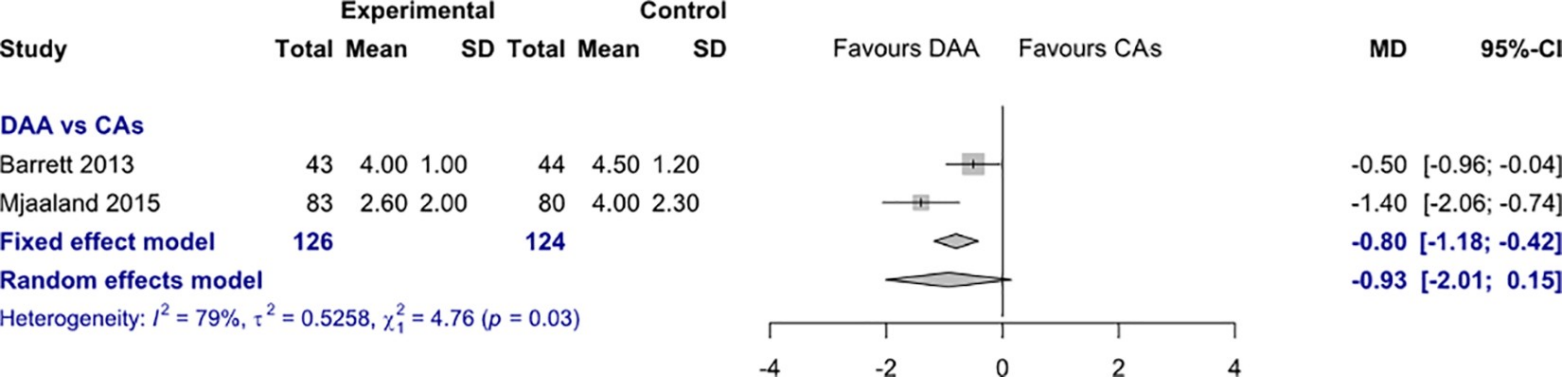
Comparison of the pain VAS 1 day postoperatively. DAA, direct anterior approach; CAs, conventional approaches; SD, standard deviation; MD, mean difference; CI, confidence interval.

*Pain VAS 1 day postoperatively*. Data on 250 patients were pooled from 2 RCTs (I^2^ = 79%, p = 0.03, Fig 5). The pain VAS 1 day postoperatively of THA through DAA was 0.8 points less than the pain VAS 1 day postoperatively of THA through CAs, using a fixed effects model (MD = -0.8, 95% CI -1.2 to -0.4). There was no difference in pain VAS 1 day postoperatively, using a random effects model (MD = -0.9, 95% CI -2.0 to 0.15).

#### 3. Functional outcome: Harris Hip Score

**Fig 6:**
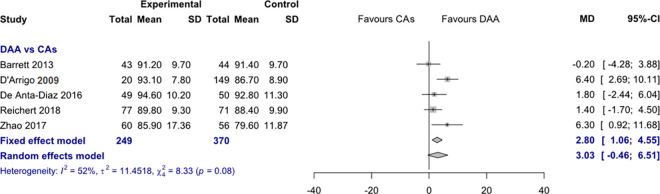
Comparison of the HHS 3 months postoperatively. DAA, direct anterior approach; CAs, conventional approaches; SD, standard deviation; MD, mean difference; CI, confidence interval.

*HHS 3 months postoperatively*. Data on 619 patients were pooled from 5 RCTs (I^2^ = 52%, p = 0.08, Fig 6). The HHS 3 months postoperatively of THA through DAA was 2.8 points higher than the HHS 3 months postoperatively of THA through CAs, using a fixed effects model (MD = 2.8, 95% CI 1.1 to 4.6). There was no difference in HHS 3 months postoperatively, using a random effects model (MD = 3.0, 95% CI -0.5 to 6.5).

**Fig 7:**
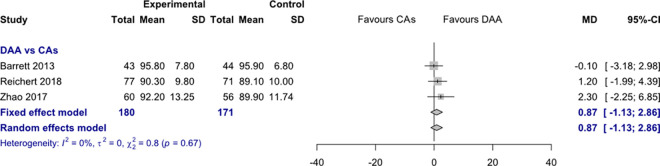
Comparison of the HHS 6 months postoperatively. DAA, direct anterior approach; CAs, conventional approaches; SD, standard deviation; MD, mean difference; CI, confidence interval.

*HHS 6 months postoperatively*. Data on 351 patients were pooled from 3 RCTs (I^2^ = 0%, p = 0.67, Fig 7). There was no difference in HHS 6 months postoperatively, using a fixed effects model (MD = 0.9, 95% CI -1.1 to 2.9) and a random effects model (MD = 0.9, 95% CI -1.1 to 2.9).

**Fig 8:**
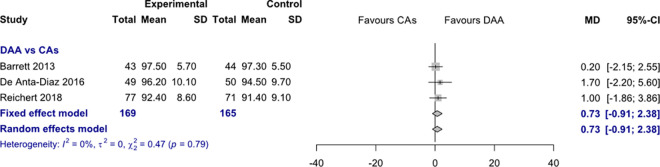
Comparison of the HHS 12 months postoperatively. DAA, direct anterior approach; CAs, conventional approaches; SD, standard deviation; MD, mean difference; CI, confidence interval.

*HHS 12 months postoperatively*. Data on 334 patients were pooled from 3 RCTs (I^2^ = 0%, p = 0.79, Fig 8). There was no difference in HHS 12 months postoperatively, using a fixed effects model (MD = 0.7, 95% CI -0.9 to 2.4) and a random effects model (MD = 0.7, 95% CI -0.9 to 2.4).

#### 4. Radiological outcome

**Fig 9:**
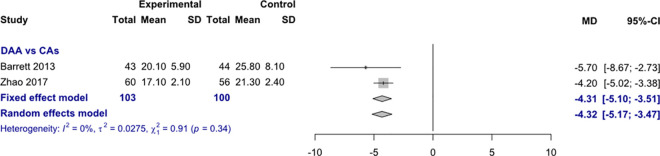
Comparison of the acetabular cup anteversion angle in degrees. DAA, direct anterior approach; CAs, conventional approaches; SD, standard deviation; MD, mean difference; CI, confidence interval.

*Acetabular cup anteversion angle*. Data on 203 patients were pooled from 2 RCTs (I^2^ = 0%, p = 0.34, Fig 9). The acetabular cup anteversion angle of THA through DAA was 4.3° lower than the acetabular cup anteversion angle of THA through CAs, using a fixed effects model (MD = -4.3, 95% CI -5.1 to -3.5). The acetabular cup anteversion angle of THA through DAA was 4.3° lower than the acetabular cup anteversion angle of THA through CAs, using a random effects model (MD = -4.3, 95% CI -5.2 to -3.5).

**Fig 10:**
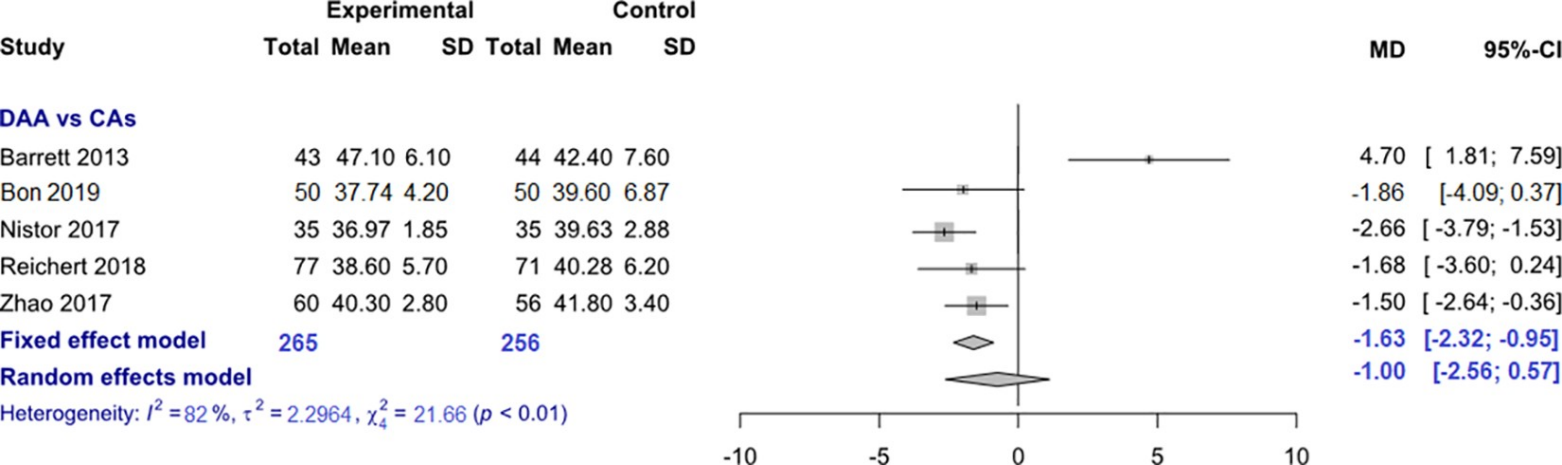
Comparison of the acetabular cup inclination angle in degrees. DAA, direct anterior approach; CAs, conventional approaches; SD, standard deviation; MD, mean difference; CI, confidence interval.

*Acetabular cup inclination angle*. Data on 521 patients were pooled from 5 RCTs (I^2^ = 82%, p<0.01, Fig 10). The acetabular cup inclination angle of THA through DAA was 1.6° lower than the acetabular cup inclination angle of THA through CAs, using a fixed effects model (MD = -1.6, 95% CI -2.3 to -0.9). There was no difference in acetabular cup inclination angle, using a random effects model (MD = -1.0, 95% CI -2.6 to 0.6).

#### Traction table subgroup analysis

A TT was used in two [56,57] out of 9 studies on THA through DAA. The utilization of TT in THA through DAA showed no influence on the overall effect of the DAA group.

#### Discussion of the main findings

We performed a direct comparison on short-term outcomes between THA through DAA and CAs. The meta-analysis was conducted using a fixed and a random effects model for all outcomes. Our meta-analysis included 9 RCTs and 998 patients. We assessed the risk of bias of the RCTs included using the Cochrane’s Risk of Bias 2 (RoB 2) tool [42]. Furthermore, we considered the publication year in risk of bias assessment [44]. Three out of 9 studies were RCTs with a low risk of bias [57,60,64], 3 out of 9 were RCTs with a moderate risk of bias [59,61,62], 3 out of 9 were RCTs with a high risk of bias [56,58,63]. We assessed the level of evidence according to the guidelines of the Centre for Evidence-Based Medicine (Oxford, UK) [43]. Three out of 9 studies were blinded RCTs with a level I evidence [57,60,64], and the other 6 studies were non-blinded RCTs with a level II evidence [56,58,59,61–63].

THA through DAA showed a longer operation time, a shorter incision length and a higher intraoperative blood loss than THA through CAs. THA through DAA showed a better pain VAS score 1 day postoperatively than THA through CAs. THA through DAA showed a higher HHS 3 months postoperatively compared to THA through CAs. The subsequent HHS 6 and 12 months postoperatively showed no difference. Both approaches showed overall sufficient results in acetabular cup positioning.

#### Overview of related meta-analyses on THA through DAA

All meta-analyses on THA through DAA had their strengths and weaknesses. The differences between them helped us to get an overall fuller picture of the comparison between THA through DAA and THA through CAs.

The 2015 meta-analysis by Higgins et al. [26] compared THA through DAA with THA through posterior approach. This meta-analysis included 17 studies with a total of 2,302 patients. Unfortunately, this meta-analysis included only 2 RCTs, 10 retrospective studies, and 5 non-randomized prospective studies. The inclusion of studies with a high risk of bias and a low level of evidence might have affected the validity of the end point estimates and overall results.

The 2019 meta-analysis by Jia et al. [27] compared THA through DAA with THA through posterior approach. This meta-analysis included 20 studies with a total of 7,377 patients. Unfortunately, this meta-analysis included only 4 RCTs, 3 prospective studies and 13 retrospective studies. The inclusion of studies with a high risk of bias and a low level of evidence might have affected the validity of the end point estimates and overall results.

The 2019 meta-analysis by Kucukdurmaz et al. [28] compared THA through DAA with THA through other approaches, including only RCTs. This meta-analysis included 18 RCTs with a total of 1,661 patients. This meta-analysis found overall more RCTs than our meta-analysis. Unfortunately, navigated THA and THA through mini-incision approaches were pooled in one group with THA through CAs. Furthermore, two relevant recent RCTs were not included in final meta-analysis [57,62].

The 2018 meta-analysis by Miller et al. [29] compared THA through DAA with THA through posterior approach. This meta-analysis included 13 prospective studies with 524 patients treated with THA through DAA and 520 patients treated with THA through posterior approach. Unfortunately, this meta-analysis included only 7 RCTs and a short follow-up period of 90 days postoperatively. Furthermore, some of the outcome parameters were pooled from studies that measured those parameters at different times. In this meta-analysis [29] a sensitivity analysis was performed which showed that the overall results remained unchanged when assessing only RCTs. Nevertheless, the same first author published a further meta-analysis two months later [65], including exactly the same 7 RCTs that were used in their sensitivity analysis before [29,65].

The 2018 meta-analysis by Putananon et al. [30] was the first attempt of a network meta-analysis to compare different surgical approaches in their outcome for THA, including only RCTs. They investigated the outcomes of THA through DAA, lateral, posterior and posterior-2 incision approaches. This network meta-analysis included 14 RCTs of THA with different approaches with a total of 1,201 patients. Putananon et al. [30] limited their investigation to three outcome parameters—VAS, HHS and postoperative complications. This meta-analysis was performed with high quality methods. It therefore has a high scientific value, although posterior and posterior-2 incision approaches were pooled based on only two studies.

The 2018 meta-analysis by Wang et al. [31] was the first meta-analysis to compare THA through DAA with THA through posterior approach, including only RCTs. This meta-analysis included 9 RCTs with a total of 377 THA through DAA and 377 THA through posterior approach. This meta-analysis was performed with high quality methods. The meta-analysis by Wang et al. [31] investigated some different outcome parameters compared to our meta-analysis. Another difference was that Wang et al. included only THA from posterior approach out of CAs. Therefore, both meta-analyses included only partially the same RCTs and they both complement each other.

The 2015 meta-analysis by Yue et al. [32] was the first meta-analysis to compare the outcomes of THA through DAA with THA through lateral approach. This meta-analysis included 12 studies with 2991 cases of THA through DAA and 1910 cases of THA through lateral approach. Unfortunately, only 2 of the studies included were RCTs and 10 were non-randomized studies. The inclusion of studies with a high risk of bias and a low level of evidence might have affected the validity of the end point estimates and overall results.

The studies by Kyriakopoulos et al. and Meermans et al. were systematic reviews without any attempt of meta-analysing the outcomes of THA through DAA [33,34].

#### Total hip arthroplasty through direct anterior approach compared to conventional approaches–trying to draw a definitive conclusion

*Operation time*. Our meta-analysis found that the operation time through DAA was 15.5 min. longer than the operation time through CAs, using a fixed effects model and 19.1 min. longer, using a random effects model. The 2015 meta-analysis by Higgins et al. [26] found no differences in operation time between THA through DAA and THA through posterior approach. The meta-analysis by Jia et al. [27] found that THA through DAA had a 13 min. longer operation time than THA through posterior approach. The meta-analysis by Kucukdurmaz et al. [28] found that THA through DAA had a 7 min. longer operation time than THA through other approaches. The meta-analysis by Miller et al. [29] did not investigate operation time. The meta-analysis by Wang et al. [31] showed no difference between THA through DAA and posterior approach in operation time. The meta-analysis by Yue et al. [32] showed that THA through DAA had an 8 min. longer operation time than THA through lateral approach. In summary, we can conclude with the following: THA through DAA had longer operation time than THA through CAs, but a similar operation time compared to THA through posterior approach.

*Incision length*. Our meta-analysis showed a 2.9 cm shorter incision length of THA through DAA than THA through CAs, using a fixed effects model. There was no difference in incision length, using a random effects model. The meta-analysis by Kucukdurmaz et al. [28] found that THA through DAA had a 3.2 cm shorter incision length than THA through other approaches. The meta-analysis by Wang et al. [31] showed a 3.5 cm shorter incision length of THA through DAA than THA through posterior approach. The meta-analyses by Higgins et al. [26], Jia et al. [27], Miller et al. [29] and Yue et al. [32] did not investigate incision length. In summary, we can conclude with the following: THA through DAA had an approximately 3.0 cm shorter incision length than THA through CAs.

*Intraoperative blood loss*. Our meta-analysis showed that the intraoperative blood loss of THA through DAA was 51.5 ml higher than the intraoperative blood loss of THA through CAs, using a fixed effects model. There was no difference in intraoperative blood loss, using a random effects model. The meta-analysis by Higgins et al. [26] showed no difference in intraoperative blood loss between THA through DAA and THA through posterior approach. The meta-analyses by Jia et al. [27], Kucukdurmaz et al. [28] and Miller et al. [29] did not investigate the blood loss of THA through DAA. The meta-analysis by Wang et al. [31] investigated the postoperative blood loss between THA through DAA and THA through posterior approach. Compared with THA through posterior approach, THA through DAA showed a reduction of the postoperative blood loss 67 ml. The meta-analysis by Yue et al. [32] showed no difference in postoperative blood transfusion rates between THA through DAA and THA through lateral approach. In summary, we can conclude that there is no relevant difference in intraoperative blood loss of THA through DAA and THA through CAs.

*VAS 1 day postoperatively*. Our meta-analysis showed a 0.8 point lower pain VAS 1 day postoperatively in THA through DAA than THA through CAs, using a fixed effects model. There was no difference in pain VAS 1 day postoperatively, using a random effects model. The meta-analysis by Higgins et al. [26] and Jia et al. [27] did not meta-analyse pain scores. The meta-analysis by Kucukdurmaz et al. [28] showed a 1.3 points lower pain VAS 1 day postoperatively for THA through DAA than THA through other approaches. The meta-analysis by Miller et al. [29] showed an overall 0.4 point lower VAS for THA through DAA than THA through posterior approach. The pain was measured at different times in a 90 days postoperative follow-up. The network meta-analysis by Putananon et al. [30] showed that THA through lateral approach had better results in pain VAS than THA through DAA, posterior and posterior-2 incision approach with 0.1, 0.6 and 1.3 points, respectively. The pain was measured at any different time according to the follow-up period of the included studies. The meta-analysis by Wang et al. [31] showed that THA through DAA had a 0.7, 1.5 and 1.6 points lower VAS at 1,2 and 3 day postoperatively than THA through posterior approach. The meta-analysis by Yue et al. [32] did not meta-analyse pain VAS postoperatively. In summary, the overall results regarding pain VAS postoperatively were very contradictive. So, we can conclude with the following: THA through DAA showed a tendency towards lower pain VAS 1 day postoperatively than THA through CAs.

*Harris Hip Score*. Our meta-analysis showed a 2.8 points higher HHS 3 months postoperatively in THA through DAA than THA through CAs, using a fixed effects model. There was no difference in HHS 3 months postoperatively, using a random effects model. The subsequent HHS 6 and 12 months postoperatively showed no difference, using a fixed and a random effects model. The meta-analysis by Higgins et al. [26] gathered information on functional outcome between THA through DAA and posterior approach from several different scores: HHS, HOOS, (Hip Disability and Osteoarthritis Outcome Score), S&R (Sports and recreation), SF (Short-Form Health Survey) WOMAC (Western Ontario and McMaster Universities Arthritis Index), Oxford Hip Score, Japanese Orthopedic Association Hip Score. The follow-up time for functional outcome varied from 1.5–3 months postoperatively. The overall results showed a weak tendency towards better outcome in THA through DAA compared to THA through posterior approach. The meta-analysis by Jia et al. [27] did not meta-analyse pain scores. The meta-analysis by Kucukdurmaz et al. [28] showed that THA through DAA had a 5.6 points higher HHS 1.5 months postoperatively than THA through other approaches. Further, this meta-analysis compared the WOMAC (Western Ontario and McMaster Universities Osteoarthritis Index) Score 1.5 months postoperatively. In this score a lower result is interpreted as better and a higher result as worse in contrast to the HHS. Kucukdurmaz et al. [28] found that THA through DAA had a 3.1 points lower WOMAC Score 1.5 months postoperatively than THA through other approaches. The meta-analysis by Miller et al. [29] showed an overall 0.3 point higher HHS for THA through DAA than THA through posterior approach. The HHS was measured at different times in a 90 days postoperative follow-up. The network meta-analysis by Putananon et al. [30] showed that THA through DAA had a better functional outcome than THA through lateral, posterior and posterior-2 incision approach. THA through DAA had a 2.6 points higher HHS 1–1.5 months postoperatively than THA through lateral approach, a 4.8 points higher HHS 1–1.5 months postoperatively than THA through posterior approach and a 10.8 higher HHS 1–1.5 months postoperatively than THA through posterior-2 incision approach. The HHS of THA through DAA, measured at the latest time in the different follow-up period of the included studies, was 6.9 points higher than THA through lateral approach, 2.4 points higher than THA through posterior approach and 4.4 higher than THA through posterior-2 incision approach. The meta-analysis by Wang et al. [31] found that THA through DAA had a 7.4 and 6.8 points higher HHS, respectively, 0.5 and 1.5 months postoperatively than THA through posterior approach. The meta-analysis by Yue et al. [32] examined several different scores (HHS, SF-36, UCLA, DAQ, WOMAC, LEFS, and LASA) describing the functional outcome. The follow-up time for functional outcome varied from 1.5 months up to years postoperatively. They stated an overall better functional outcome of THA through DAA compared to THA through lateral approach. In summary, we can conclude with the following: THA through DAA had a better early (up to 3 months) postoperative functional outcome than THA through CAs. More quality RCTs and meta-analyses are required to draw a conclusion regarding subsequent postoperative functional outcome.

*Acetabular cup positioning*. The acetabular cup anteversion angle of THA through DAA was 4.3° lower than the acetabular cup anteversion angle of THA through CAs, using a fixed and random effects model. The acetabular cup inclination angle of THA through DAA was 1.6° lower than the acetabular cup inclination angle of THA through CAs, using a fixed effects model. There was no difference in acetabular cup inclination angle, using a random effects model. The acetabular cup anteversion angle varied in the RCTs included in our meta-analysis from 17.1°-20.1° in THA through DAA and the acetabular cup inclination angle varied from 37°-47.1° in THA through DAA. Since the ideal values for acetabular cup positioning vary for inclination from 40° to 50° and for anteversion from 10° to 25° [40], THA through DAA obviously showed a slight tendency towards a too flat inclination angle. The meta-analysis by Higgins et al. [26] showed no differences in acetabular cup positioning between THA through DAA and THA through posterior approach. The meta-analysis by Jia et al. [27] found no difference in acetabular cup inclination angle between THA through DAA and THA through posterior approach. Jia et al. found a 3.8° lower acetabular cup inclination angle of THA through DAA than THA through posterior approach. The meta-analyses by Kucukdurmaz et al. [28], Miller et al. [29] and Wang et al. [31] did not investigate radiological outcomes. The meta-analysis by Yue et al. [32] found no difference in acetabular cup inclination and anteversion angles between THA through DAA and THA through lateral approach. The acetabular cup anteversion angle varied from 21.8°-24° degrees in THA through DAA and the acetabular cup inclination angle varied from 37.6°-47° in THA through DAA. Analysing the acetabular cup anteversion angle, we have to emphasize that this is a very questionable outcome parameter since this angle was measured in almost every study in conventional radiographs. The acetabular cup anteversion angle can only be measured reliably with a CT-scan [66]. In summary, we can conclude that THA through DAA showed sufficient results in acetabulum cup positioning with a slight tendency towards a too flat inclination angle.

*Postoperative complications*. In our meta-analysis we could not include enough data on postoperative complications such as dislocation, infection, periprosthetic fracture, pulmonary embolism, infection, wound healing problems and heterotopic ossifications. Nevertheless, this is a very important outcome parameter and it was interesting what other meta-analyses had found so far. The meta-analysis by Higgins et al. [26] showed better results in postoperative dislocation for THA through DAA than THA through CAs. The meta-analysis by Jia et al. [27] investigated the following postoperative complications: dislocation, lateral cutaneous nerve of the thigh neuropraxia, intraoperative fractures. This meta-analysis found that THA through DAA had worse results in lateral cutaneous nerve of the thigh neuropraxia and intraoperative fractures than THA through posterior approach. The meta-analysis by Kucukdurmaz et al. [28] investigated the following postoperative complications: infection, wound healing problems, neurovascular damage, fracture, thrombosis, dislocation, component malpositioning, heterotopic ossification and death. This meta-analysis found no difference in the overall complication rates between THA through DAA and THA through other approaches. The meta-analysis by Miller et al. [29] investigated the following postoperative complications over 90 days of follow-up: dislocation, fracture, hematoma, infection, thromboembolic event, and reoperation. This meta-analysis found no differences in postoperative complication between THA through DAA and THA through posterior approach. The network meta-analysis by Putananon et al. [30] compared the following postoperative complications in THA through DAA, lateral, posterior and posterior-2 incision approaches: dislocation, infection and fracture. This meta-analysis showed better results for THA through posterior approach followed by THA through DAA, lateral and posterior-2 incision approaches. The meta-analysis by Wang et al. [31] showed no differences between THA through DAA and THA through posterior approach in terms of intraoperative fracture, postoperative dislocation, and heterotopic ossification. The meta-analysis by Yue et al. [32] showed that no differences between THA through DAA and THA through lateral approach in terms of dislocation, intraoperative fracture, superficial infection, deep infection, and postoperative hematoma. This meta-analysis found that THA through DAA had a higher risk of lateral cutaneous nerve of the thigh palsy. More quality RCTs and meta-analyses are required to draw a conclusion regarding postoperative complications. So far we can conclude that THA through DAA had a higher risk of damage of the lateral cutaneous nerve of the thigh.

*Traction table utilization*. In subgroup analysis our study showed that there was no influence of TT utilization on outcomes in THA through DAA. A recent 2020 systematic review on DAA by Sarraj included 44 studies with a total of 26,353 patients [67]. The study found no relevant difference in outcome between TT versus standard table THA through DAA.

### Limitations

We identified the following limitations to our study: First, the long-term outcomes in THA were not considered. Second, due to insufficient data, important outcome parameters such as hospitalization time, postoperative drainage volume and postoperative complications could not be considered. Third, we did not consider the possible influence of the surgeon operating skills, the utilization of tranexamic acid and anticoagulants, bone cement or the types of implants for hip replacement. Fourth, our findings include THA as both elective and traumatic surgery. Fifth, we excluded studies comparing DAA with mini-incision approaches. The point behind our intention in excluding mini-incision approaches was to enable a clear comparison between a purely minimally invasive DAA and purely conventional approaches. Nevertheless, this might have influenced our findings. Sixth, our meta-analysis did not include enough studies to assess publication bias tests and plots [68], which present a major limitation to our study. Lastly, CAs were summarized in one group, although they differ greatly from one another.

## Conclusions

Considering the results of our meta-analysis and the review of related meta-analyses, we can conclude that short-term outcomes of THA through DAA were overall better than THA through CAs. THA through DAA had shorter incision length, a tendency towards lower pain VAS 1 day postoperatively and better early (up to 3 months) postoperative functional outcome than THA through CAs. The intraoperative blood loss showed indifferent results. THA through DAA had a longer operation time than THA through CAs.

## Supporting information

S1 Checklist PRISMA 2009 checklist. (DOC)

S1 Appendix (DOCX)

S1 Dataset (XLSX)

## Abbreviations

CI: confidence intervals
CA: conventional approach
DAA: direct anterior approach
HHS: Harris hip score
MD: mean difference
SMD: standardized mean difference
RCT: randomized controlled trial
RoB: risk of bias
RR: risk ratio
THA: total hip arthroplasty
TT: traction table
VAS: Visual Analogue Scale
WOMAC: Western Ontario and McMaster Universities Arthritis Index
