# Expression of Concern: Direct anterior approach (DAA) vs. conventional approaches in total hip arthroplasty: A RCT meta-analysis with an overview of related meta-analyses

**DOI:** 10.1371/journal.pone.0283509

**Published:** 2023-03-20

**Authors:** The PLOS ONE Editors

After publication of this article [1], concerns were raised about possible redundant publication across a group of closely related publications [1–7].

PLOS investigated this matter and confirmed that the articles published in [1] and [2] addressed the same primary research question and were submitted within a short time of one another. The *PLOS ONE* article [1] was published before the *Scientific Reports* article [2].

Studies [3, 6–7] and the studies published previously in [4–5] addressed closely related research questions but included a different treatment group than [1, 2] and in the case of [3, 5–7] used a different methodology.

The following issues were noted with regards to [1] and [2]:

- The two articles referenced the same PROSPERO protocol and reported largely overlapping studies and results, with the exception that [2] included an additional study.
- According to [2], an updated literature search was performed before the submission of article [1]. It is not clear why the updated dataset was not included in [1].
- Despite the overlap between [1, 2], the articles report different conclusions regarding the relative outcomes of THA through DAA compared to THA through CAs. Although this could be explained by effects of the additional study included in [2], this discrepancy raises concerns about the reliability and robustness of the *PLOS ONE* article’s [1] conclusion.
- As a closely related study, the submission of article [2] was not declared during the submission of article [1] as is required by PLOS’ Policy on Submission and Publication of Related Studies.

In light of these concerns, the *PLOS ONE* Editors issue this Expression of Concern.
