# Supplementary material for: Direct anterior approach (DAA) vs. conventional approaches in total hip arthroplasty: A RCT meta-analysis with an overview of related meta-analyses
Source: PLoS One. 2021 Aug 24;16(8):e0255888. doi: 10.1371/journal.pone.0255888 (PMC8384214; doi:10.1371/journal.pone.0255888)
Supplement: S1 Appendix — (DOCX) [file pone.0255888.s002.docx]

Appendix :

I. Search strategy **PubMed:**

((DAA) OR (direct anterior approach)) ti,ab.

II. Search strategy **Cochrane Library**:

((DAA) OR (direct anterior approach)) in Title Abstract Keyword

III. Search Strategy **Google Scholar**:

(direct anterior approach)

IV. Search strategy **Clinical Trials:**

(DAA) OR (direct anterior approach)
